# Supplementary material for: Comparison of Secular Trends in Esophageal Cancer Mortality in China and Japan during 1990–2019: An Age-Period-Cohort Analysis
Source: Int J Environ Res Public Health. 2022 Aug 18;19(16):10302. doi: 10.3390/ijerph191610302 (PMC9408772; doi:10.3390/ijerph191610302)
Supplement: Supplementary file 1 [file ijerph-19-10302-s001.zip › ijerph-1857291-supplementary.pdf]

Supplementary Materials

Table S1. Mortality rate of esophageal cancer by age, period, and median birth cohorts among men in China, 1990 to 2019 (per 100 000)

**Median period**	**Age group**												**Median birth cohort**
	25-29	30-34	35-39	40-44	45-49	50-54	55-59	60-64	65-69	70-74	75-79	80-84	
China(male)												267.62	1912
											245.81	272.19	1917
										211.82	245.29	322.35	1922
									150.85	205.32	278.54	314.71	1927
								103.53	140.39	224.11	248.53	255.85	1932
							71.78	97.25	152.08	199.96	207.61	225.20	1937
						39.89	66.47	105.14	136.21	158.13	183.93		1942
					20.48	40.32	76.33	103.06	126.48	147.45			1947
				10.91	20.04	46.51	69.74	87.24	99.82				1952
			2.90	10.43	23.10	42.00	51.73	69.59					1957
		0.85	2.60	10.56	18.65	27.32	41.32						1962
	0.22	0.77	3.03	9.70	14.86	22.31							1967
1992	0.21	0.90	2.76	5.61	10.56								1972
1997	0.23	0.78	1.71	4.70									1977
2002	0.20	0.65	1.61										1982
2007	0.18	0.58											1987
2012	0.14												1992
2017													

Table S2. Mortality rate of esophageal cancer by age, period, and median birth cohorts among women in China, 1990 to 2019 (per 100 000)

**Median period**	**Age group**												**Median birth cohort**
	25-29	30-34	35-39	40-44	45-49	50-54	55-59	60-64	65-69	70-74	75-79	80-84	
China(female)												149.13	1912
											139.72	143.00	1917
										115.01	134.17	164.54	1922
									81.24	109.82	144.45	154.88	1927
								48.09	71.84	108.79	116.72	113.32	1932
							26.59	41.08	71.28	84.77	79.64	87.00	1937
						12.22	19.47	42.75	52.93	55.67	64.86		1942
					4.90	12.21	23.65	34.26	35.52	47.11			1947
				2.43	6.23	13.06	17.96	19.82	25.67				1952
			0.76	2.43	5.70	8.87	9.37	13.90					1957
		0.31	0.74	2.21	3.03	3.83	6.41						1962
	0.15	0.46	0.76	1.58	1.78	2.71							1967
1992	0.24	0.39	0.54	0.79	1.18								1972
1997	0.19	0.22	0.30	0.55									1977
2002	0.11	0.14	0.23										1982
2007	0.08	0.11											1987
2012	0.06												1992
2017													

Table S3. Mortality rate of esophageal cancer by age, period, and median birth cohorts among men in Japan, 1990 to 2019 (per 100 000)

**Median period**	**Age group**												**Median birth cohort**
	25-29	30-34	35-39	40-44	45-49	50-54	55-59	60-64	65-69	70-74	75-79	80-84	
Japan(male)												87.15	1912
											74.38	83.47	1917
										61.35	72.40	80.46	1922
									50.25	60.76	72.48	80.14	1927
								38.31	51.53	62.67	74.05	81.91	1932
							24.36	36.96	50.26	61.08	70.70	76.83	1937
						12.18	23.49	36.37	49.07	58.53	65.88		1942
					5.27	12.63	23.73	35.54	46.26	56.32			1947
				1.50	5.10	11.48	20.96	30.79	39.93				1952
			0.32	1.35	4.42	9.57	17.08	25.72					1957
		0.09	0.28	1.20	3.48	7.63	14.31						1962
	0.03	0.09	0.26	0.98	2.64	6.10							1967
1992	0.03	0.09	0.22	0.82	2.19								1972
1997	0.03	0.08	0.21	0.73									1977
2002	0.02	0.07	0.19										1982
2007	0.02	0.07											1987
2012	0.02												1992
2017													

Table S4. Mortality rate of esophageal cancer by age, period, and median birth cohorts among women in Japan, 1990 to 2019 (per 100 000)

**Median period**	**Age group**												**Median birth cohort**
	25-29	30-34	35-39	40-44	45-49	50-54	55-59	60-64	65-69	70-74	75-79	80-84	
Japan(female)												21.72	1912
											14.05	18.05	1917
										8.43	11.57	14.91	1922
									5.24	6.93	9.52	12.68	1927
								3.54	4.68	6.45	8.55	11.67	1932
							2.42	3.61	4.73	6.38	8.36	11.05	1937
						1.38	2.54	3.83	4.71	6.30	8.23		1942
					0.64	1.54	2.71	3.77	4.81	6.28			1947
				0.27	0.69	1.64	2.70	3.69	4.60				1952
			0.09	0.28	0.68	1.63	2.48	3.44					1957
		0.03	0.09	0.28	0.69	1.57	2.30						1962
	0.02	0.03	0.09	0.29	0.66	1.49							1967
1992	0.02	0.03	0.09	0.29	0.63								1972
1997	0.02	0.03	0.08	0.28									1977
2002	0.02	0.03	0.08										1982
2007	0.01	0.02											1987
2012	0.01												1992
2017													

Table S5. Estimated coefficients for age, period, and cohort effects on esophageal cancer mortality.

	China				Japan			
	Men		Women		Men		Women	
**Factor**	**Coefficient**	**95%CI**[a]	**Coefficient**	**95%CI**	**Coefficient**	**95%CI**	**Coefficient**	**95%CI**
**Age**								
25-29	-1.21	-1.45 to -0.97	-0.21	-0.56 to 0.14	-1.10	-1.37 to -0.83	-0.69	-1.05 to -0.33
30-34	-0.48	-0.64 to -0.33	-0.21	-0.49 to 0.08	-0.64	-0.82 to -0.46	-0.74	-1.03 to -0.44
35-39	0.02	-0.09 to 0.13	-0.28	-0.51 to -0.05	-0.35	-0.49 to -0.22	-0.23	-0.44 to -0.03
40-44	0.59	0.50 to 0.67	0.06	-0.11 to 0.23	0.35	0.26 to 0.44	0.34	0.19 to 0.49
45-49	0.66	0.59 to 0.73	0.19	0.05 to 0.32	0.81	0.74 to 0.88	0.59	0.48 to 0.71
50-54	0.70	0.65 to 0.75	0.30	0.20 to 0.41	0.97	0.92 to 1.02	0.83	0.75 to 0.92
55-59	0.61	0.57 to 0.65	0.29	0.21 to 0.37	0.93	0.90 to 0.97	0.73	0.67 to 0.79
60-64	0.41	0.38 to 0.44	0.31	0.25 to 0.36	0.69	0.67 to 0.71	0.48	0.44 to 0.52
65-69	0.15	0.13 to 0.17	0.22	0.18 to 0.26	0.32	0.31 to 0.33	0.13	0.10 to 0.16
70-74	-0.10	-0.12 to -0.07	0.07	0.03 to 0.12	-0.14	-0.16 to -0.12	-0.17	-0.21 to -0.14
75-79	-0.47	-0.51 to -0.43	-0.21	-0.27 to -0.14	-0.64	-0.68 to -0.60	-0.48	-0.54 to -0.42
80-84	-0.87	-0.93 to -0.82	-0.54	-0.63 to -0.45	-1.19	-1.25 to -1.14	-0.80	-0.88 to -0.72
**Period**								
1990-1994	-0.08	-0.09 to -0.06	-0.11	-0.14 to -0.08	-0.02	-0.03 to -0.01	-0.02	-0.04 to 0.00
1995-1999	-0.05	-0.07 to -0.03	-0.04	-0.08 to -0.01	0.01	0.00 to 0.02	0.00	-0.02 to 0.02
2000-2004	0.12	0.10 to 0.14	0.17	0.13 to 0.20	0.02	0.01 to 0.03	0.02	0.00 to 0.04
2005-2009	0.11	0.10 to 0.13	0.14	0.10 to 0.17	0.01	0.01 to 0.02	0.02	0.00 to 0.04
2010-2014	-0.02	-0.04 to -0.01	-0.07	-0.11 to -0.03	0.00	-0.01 to 0.00	0.01	-0.01 to 0.03
2015-2019	-0.09	-0.10 to -0.07	-0.08	-0.12 to -0.05	-0.01	-0.02 to -0.01	-0.02	-0.04 to -0.01
**Cohort**								
1910-1914	-0.31	-0.44 to -0.19	-0.66	-0.87 to -0.46	-0.28	-0.37 to -0.19	0.45	0.32 to 0.58
1915-1919	-0.18	-0.26 to -0.11	-0.44	-0.59 to -0.29	-0.24	-0.31 to -0.17	0.30	0.20 to 0.41
1920-1924	-0.08	-0.14 to -0.03	-0.24	-0.35 to -0.13	-0.19	-0.25 to -0.14	0.12	0.04 to 0.20
1925-1930	-0.02	-0.06 to 0.02	-0.06	-0.15 to 0.02	-0.11	-0.15 to -0.07	-0.04	-0.10 to 0.02
1931-1934	0.00	-0.04 to 0.03	0.03	-0.03 to 0.09	0.00	-0.02 to 0.03	-0.12	-0.16 to -0.07
1935-1939	0.02	-0.01 to 0.04	0.09	0.05 to 0.14	0.06	0.05 to 0.08	-0.11	-0.14 to -0.08
1940-1944	0.03	0.01 to 0.05	0.12	0.07 to 0.17	0.12	0.11 to 0.13	-0.08	-0.11 to -0.05
1945-1949	0.10	0.08 to 0.13	0.20	0.14 to 0.27	0.19	0.17 to 0.20	-0.04	-0.08 to 0.00
1950-1954	0.13	0.09 to 0.16	0.22	0.13 to 0.31	0.16	0.13 to 0.19	-0.01	-0.07 to 0.04
1955-1959	0.11	0.07 to 0.16	0.17	0.05 to 0.28	0.10	0.06 to 0.15	-0.01	-0.09 to 0.07
1960-1964	0.02	-0.04 to 0.08	-0.01	-0.17 to 0.15	0.01	-0.05 to 0.07	0.00	-0.11 to 0.10
1965-1969	0.00	-0.07 to 0.07	-0.06	-0.25 to 0.12	-0.12	-0.19 to -0.04	0.04	-0.09 to 0.16

	China				Japan			
	Men		Women		Men		Women	
Factor	Coefficient	95%CI[a]	Coefficient	95%CI	Coefficient	95%CI	Coefficient	95%CI
1970-1974	-0.11	-0.20 to -0.02	-0.09	-0.33 to 0.15	-0.18	-0.28 to -0.09	0.04	-0.12 to 0.20
1975-1979	-0.18	-0.31 to -0.05	-0.14	-0.50 to 0.21	-0.10	-0.23 to 0.04	0.09	-0.13 to 0.30
1980-1984	-0.07	-0.28 to 0.15	-0.15	-0.73 to 0.42	-0.04	-0.29 to 0.20	0.04	-0.34 to 0.42
1985-1989	0.02	-0.35 to 0.39	-0.20	-1.06 to 0.67	0.02	-0.43 to 0.46	0.01	-0.70 to 0.71
1990-1994	0.00	-0.85 to 0.86	-0.18	-1.75 to 1.40	0.02	-1.02 to 1.06	-0.03	-1.36 to 1.31

Note: [a]95%CI is the abbreviation of the 95% confidence interval

Table S6. Wald Chi-Square tests for estimable functions in the APC model.

Null Hypothesis	China				Japan			
	Males		Females		Males		Females	
	Chi-Square	*P*-Value	Chi-Square	*P*-Value	Chi-Square	*P*-Value	Chi-Square	*P*-Value
**Net Drift = 0**	255.54	<0.001	336.56	<0.001	187.00	<0.001	5.48	<0.05
**All Period RR = 1**	720.65	<0.001	535.94	<0.001	247.71	<0.001	17.53	<0.05
**All Cohort RR = 1**	1039.21	<0.001	1661.84	<0.001	2863.68	<0.001	918.86	<0.001
**All Local Drifts = Net Drift**	130.99	<0.001	126.40	<0.001	1289.38	<0.001	415.24	<0.001

Notes: Net drifts refer to the overall annual percentage change in the age-standardized rate. Local drifts reflect the annual percentage change of age-specific mortality. All net drifts and local drifts were statistically significant ($p < 0.05$)
